# Ehlers-Danlos Syndrome in the Field of Psychiatry: A Review

**DOI:** 10.3389/fpsyt.2021.803898

**Published:** 2022-01-11

**Authors:** Hiroki Ishiguro, Hideaki Yagasaki, Yasue Horiuchi

**Affiliations:** ^1^Center of Genetic Medicine, Hospital, University of Yamanashi, Kofu, Japan; ^2^Cancer Counseling and Support Center, Hospital, University of Yamanashi, Kofu, Japan; ^3^Department of Neuropsychiatry, Graduate School of Medicine, University of Yamanashi, Kofu, Japan; ^4^Department of Psychiatry and Behavioral Sciences, Tokyo Metropolitan Institute of Medical Science, Tokyo, Japan

**Keywords:** Ehlers Danlos syndrome, pain, self-esteem, psychiatric disorders, psychiatrist, clinical geneticist, psychotropic drugs, counseling

## Abstract

Ehlers-Danlos syndrome (EDS) comprises a series of rare hereditary connective tissue diseases characterized by joint hypermobility, joint dislocation, and hyperextensibility of the skin, as well as cardiovascular involvement. EDS is often associated with chronic widespread physical pain, which can lead to psychological pain. Poor awareness and limited diagnosis of EDS and related symptoms result in decreased self-esteem and confusion regarding physical sensation. Furthermore, EDS imposes substantial psychological burden on patients due to exercise restriction, scars, keloids, and subcutaneous fat accumulation on the extremities, which leads to parental overprotection and bullying experiences from other children at school age. Recent large-scale studies have suggested that patients with EDS have a higher risk of mood disorders than the general population. Other cohort studies indicated high prevalence of anorexia nervosa, addiction, obsessive compulsive disorder, and anxiety disorder were found in patients with EDS. Case reports instead indicated that some psychiatric disorders were secondary symptoms due to physical problems from EDS. Therefore, psychiatrists must be more knowledgeable and proactive about EDS in their practice. We review the previous case reports and literature for patients with EDS, along with our own case of complicated psychiatric problems, which are strongly related to early stressful situations through childhood and adolescence. This is to aid general psychiatrists in the discussion of appropriate medical management in such infrequent, yet challenging conditions.

## Introduction

Ehlers-Danlos syndrome (EDS) comprises a series of hereditary connective tissue diseases, which is often characterized by musculoskeletal, dermatological, and cardiovascular problems. While EDS had been classified into more than 10 types in the past, it was reclassified into 13 types in 2017 based on symptomatic features: Classical (cEDS), Classical-like (clEDS), Cardiac-vascular (cvEDS), Vascular (vEDS), Hypermobile (hEDS), Arthrochalasia (aEDS), Dermatosparaxis (dEDS), Kyphoscoliotic (kEDS), Brittle Cornea syndrome (BCS), Spondylodysplastic (spEDS), Musculocontractural (mcEDS), Myopathic (mEDS), and Periodontal (pEDS) (1). EDS has an overall prevalence of 1/5,000, and multiple causative genes involved in each type have been identified, although some implicated genes remain elusive (2). The inherited form is mostly autosomal dominant in the classical, hypermobile, and arthrochalasia types, while it is autosomal recessive in the dermatosparaxis and kyphoscoliotic types. EDS mainly involves physical symptoms, and there is little evidence of central nervous system involvement in affected patients.

Although it has been established that joint hypermobility syndrome (JHS) is a connective tissue disorder that primarily affects the musculoskeletal system, while EDS comprises connective tissue disorders with multisystem manifestations, the 2017 International Classification of the EDS replaced previous terms for symptomatic joint hypermobility with hypermobile EDS, since there was a lack of clinical distinction between the hEDS (3, 4). Thus, the term JHS is no longer used according to Ehlers-Danlos Support UK (https://www.ehlers-danlos.org/what-is-eds/information-on-eds/types-of-eds/). EDS are the most common hereditary, non-inflammatory disorders of connective tissues, and the hypermobile type of EDS (hEDS) may present with common symptoms comparable to joint hypermobility, including joint pain, swelling, instability, dislocation, and back pain (3, 4).

Many psychiatrists are unfamiliar with the medical care of genetic disorders, as they especially avoid the despairing history of discrimination against mentally ill persons under the former Eugenic Protection Law. However, many clients with genetic and chromosomal diseases are likely to have mental problems, including neurodevelopmental, anxiety, and mood disorders. Some of these may occur due to congenital brain mechanisms and various socio-psychological burdens in the life of patients with EDS. Thus, doctors in Japan who are both certified as clinical geneticists and psychiatrists have the opportunity to treat patients with EDS complicated by mental disorders. By listening to their complaints and understanding their psychological problems, we believe that more psychiatrists should cooperate in the treatment and support of such patients.

Chronic pain is a common symptom of EDS, which damages the quality of life and causes psychological disability (5, 6). The combination of anxiety, depression, and pain can be thought of as an alternate interaction of physical and emotional distress in humans (7–9) and in rodents (10). Therefore, patients with EDS often experience anxiety and depression caused by chronic pain, as well as stress from surrounding incomprehension regarding their physical restrictions and social disability. A history of recurrent joint dislocation and major bleeding poses a fear in the daily lives of patients with EDS. EDS has a huge impact on physical and psychological development. Many patients have several cognitive distortions regarding themselves due to the lack of early psychological support. In this review, we summarize the clinical psychiatric endophenotypes caused by and from hEDS, as many of them have been more or less neglected in the past.

## Neurodevelopment Disorders and EDS

The current EDS classification (major types I–VII) does not include specific disease types associated with neurodevelopmental disorders. The older classification (in the 1990s and thereafter) included a certain subtype with intellectual developmental disorder. However, few evidences reported genetic factors could explain brain hypofunction and physical symptoms in EDS. Wang et al. performed whole-genome low-coverage sequencing and medical exome sequencing and found a case with *B4GALT7* gene variants associated with spondylodysplastic-type spEDS. The common symptoms of this type of EDS include progressive short stature from childhood, hypotonia, and bowing of limbs, and some minor symptoms include skin hyperextensibility, pes planus, delayed motor development, and mild intellectual disabilities (11). One of the patients we reported on (12) had moderate intellectual disabilities and did not have some of the clinical symptoms, such as short stature and sparse scalp hair and eyebrows. Therefore, we diagnosed his condition as cEDS. As EDS is a rare disease, when patients with EDS present neurodevelopmental disorders, it is difficult to distinguish the inevitable symptoms or coincidences without proof of genetic findings that could explain both physiological EDS symptoms and CNS symptoms.

Children with hEDS have a high rate of developmental coordination disorder (DCD) with learning disability and attention deficit hyperactive disorder (ADHD), which can affect their well-being and the development of the nervous system. Further clinical research is necessary to explore the pathogenesis and management of patients with EDS (13–15). Patients with hEDS may favor behavioral atypism with both hypoactivity and hyperactivity. Some patients use endurance to cope with pain, which is persistence in an unhealthily high level of activity despite pain. In addition, pain disrupts the attentional performance. These psychological reactions may contribute to ADHD (16). A high prevalence of ADHD and autism spectrum disorder (ASD) has been found in patients with hEDS (17). However, whether the prevalence of ADHD in patients with hEDS is higher than that in the general population is inconclusive because the study did not have a matched control group (18). However, another study reported that ADHD was significantly enriched in the hypermobility spectrum disorders but not in the EDS group than in general population (19). Casanova et al. suggested comorbidity and familial co-occurrence between EDS and autism (as defined by the authors) (20). Baeza-Velasco et al. reported the overrepresentation of ASD in patients with EDS (21). Regarding the biological interaction between hEDS and ASD, elevation of serum tyrosine and hydroxyproline levels in patients with ASD may provide evidence for a link between them, considering the association between hydroxyproline levels and collagen damage (22). Although shared clinical features and phenotypes between EDS and ASD are not rare, we need to specify the possible common causative genetic factors for both disorders to arrive at a conclusion.

On the contrary, few studies have reported specific learning disorders, such as dyslexia, dysgraphia, and dyscalculia, in patients with EDS, and these aspects are poorly explored. Baeza-Velasco recently summarized that physical disabilities in patients with hEDS could increase learning and communication disorders from an early age (16). The diagnosis of learning disorders is difficult because of the overlap with several EDS criteria associated with proprioception and pronounced fatigue. However, child and adolescent psychiatrists should be aware of the importance of identifying and handling these comorbidities when they exist. Notably, children with hEDS may need to be routinely screened for neuropsychiatric symptoms, such as specific learning disorders.

## Anorexia Nervosa and EDS

With regard to eating disorders in hEDS, growing clinical evidence has been reported in the previous years. hEDS presents with gastrointestinal problems, temporomandibular disorders, and smell and taste abnormalities, thereby producing significant implications for eating, which are commonly experienced by those affected (23). There are two reported cases of hEDS and anorexia nervosa complications (24, 25). Although Miles et al. reported their case as a coincidental event (25), Lee and Strand discussed in detail how patients with EDS can develop anorexia nervosa. The patient suffered from various recurrent gastrointestinal complaints, such as diffuse pain, bloating, and nausea, for ~10 years. Such gastrointestinal symptoms, in addition to persistent fatigue, muscle cramps, and joint pain, can primarily be attributed to anorexia nervosa (24). Indeed, gastrointestinal symptoms and esophagitis are very common in patients with hEDS, resulting in nausea and vomiting (26–28). Dysphagia, fragility of the oral mucosa, and smell and taste abnormalities as EDS complications, can lead to food selectivity and disordered eating patterns (23).

In addition to the psychological causes, we encountered a Japanese woman with EDS, who had anorexia nervosa (restriction type), in addition to dissociative identity disorder, gender dysphoria, and dysthymia (12). She had classical EDS symptoms with skin hyperextensibility and atrophic scarring, generalized joint hypermobility, easy bruising, soft and doughy skin, skin fragility, traumatic splitting, and pain, which is a complication of joint hypermobility. The patient was brought up as an older sister in contrast to another twin sister, although they were monozygotic twins. The younger sister had EDS, however, she had no psychiatric symptoms. A meta-analysis revealed that being victimized by bullying and teasing is a risk factor that contributed to eating disorders (29, 30); hence, the patient was likely to develop anorexia nervosa by being frequently bullied for her facial scars caused by EDS at elementary school age. In addition, we have to focus on the assumption that her anorexic symptoms in this case could have been influenced by insufficient maternal separation from early childhood, on account of her mother's overprotectiveness and excessive interference driven by her concern for the high risk of trauma associated with EDS (31, 32).

## Addiction and EDS

Since chronic pain is highly observed in patients with EDS, research should be focused on pain relief for these patients. A study indicated that opioid use among children was double in an EDS cohort compared to a control group (27.5 vs. 13.5%), while in adults, it was nearly double in patients with EDS (62 vs. 34.1%) (33). However, to the best of our knowledge, there has been no research on the misuse of analgesics among patients with EDS. While increases in tobacco and alcohol use were found in hEDS (34), not much is known about the ratio of their misuse in the EDS population, although patients are thought to use both substances to cope with distress and anxiety.

## Anxiety Disorders and EDS

A high prevalence of anxiety was found in patients with EDS (35–37). Similar to EDS, panic disorder is also found more frequently in patients with hEDS as reported by recent research and meta-analysis (38–40), while others have reported a different result (41).

In a study by Pasquini et al. symptom severity was assessed using the Hamilton Anxiety Rating Scale (HAM-A) and the Hamilton Depression Rating Scale (HAM-D) in an hEDS patient group and healthy controls. The study indicated higher scores of HAM-D and HAM-A in the hEDS patient group than in the controls (42). More clinical evidence is needed to determine what types of anxiety or anxiety-related disorders may be associated with hEDS. Based on the Symptom Check List revised 90 (SCL-90-R) that is a widely used self-report measure assessing the severity of current psychological symptoms and distress, high percentage of patients with EDS reported their somatization and obsessive-compulsivity. Severe levels of those symptoms are observed more than depressiveness, general anxiety, hostility, and phobic anxiety (43). We have been treating a patient with clEDS, due to a homozygous null mutation in the *TNXB* gene. It phenotypically resembles the classical form of EDS, although it lacks atrophic scarring (44). As an example of somatization, the patient is suffering from general pain, part of which cannot be explained by joint dislocation, internal bleeding, and other physiological damages; however, it is enhanced by anxiety caused by incomprehension from family members and surroundings regarding EDS symptoms.

## Mood Disorders, Schizophrenia, and EDS

Depressive symptoms in patients with EDS were first reported in 2003 (45). Moreover, patients with EDS are at a relatively high risk of mental disorders, such as mood disorders, and suicidal behavior accompanied by pain, including headache, muscle pain, neuralgia, abdominal pain, and malaise (17, 37, 46–50). The overall use of psychotropic drugs to treat these conditions was 41.4% in patients with EDS compared with 13.9% in controls (37).

Since patients with depression frequently experience pain (51, 52), some of the pain reported by patients with EDS could be either attributable to physical symptoms, due to dislocation or depression. Additionally, chronic pain in such patients may lead to negative emotions due to the psychological burden (47). Furthermore, a large-scale study on a Swedish population found that patients with EDS have a higher risk of mood and developmental disorders than that of the general population (17).

Tenascin-X (TNX) deficiency had been well-reported to be associated with EDS (53–60). While a single nucleotide polymorphism in *TNX* gene had been indicated to be associated with schizophrenia by Tochigi (61), the replication studies from other researchers could not find the association (62, 63). To the best of our knowledge, there has been no research on high prevalence of the schizophrenia among patients with EDS.

## Personality Disorder and EDS

The patients with hEDS had obsessive-compulsive personality disorder (OCPD) with an observed prevalence rate of >10% (42). With regard to other personality disorders, borderline personality disorder along with depression, emotionally unstable personality disorder was observed in each patient with EDS (49, 64). However, there is still not much information about personality disorders that are common in EDS.

## Psychiatric Treatment

As summarized above, it has been found that various psychiatric disorders can coexist either biologically or psychologically with EDS (Table 1). However, there is minimal information regarding the management of treatment-resistant psychiatric symptoms in patients with EDS. Therefore, we have discussed some successful management strategies for anxiety in patients with EDS who use psychotropic drugs. Since some chronic pain symptoms may be aggravated by anxiety, therapeutic alliances with patients' physicians may allow for better coping strategies for pain. Drug therapy may be effective in addition to supportive psychotherapy. Benzodiazepines are useful in managing acute anxiety symptoms and pain relief. However, if drugs are used for a longer period to treat chronic pain, patients are at a risk of dependence, withdrawal, and cognitive changes (65); therefore, information on alternative medicines is needed. Niedt et al. reported the efficacy of risperidone in patients with anxiety symptoms (66).

**Table 1:** Neuropsychiatric disorders associated with EDS.

**Disease**	**References**
Neurodevelopment disorders	(11–22)
Anorexia nervosa	(12, 23–30)
Addiction	(33, 34)
Anxiety disorders	(35–43)
Mood disorders	(17, 37, 45–50)
Personality disorder	(42, 49, 64)

A patient with borderline personality disorder reported a reduction in suicidal ideation after taking selective serotonin reuptake inhibitor and lithium as its enhancer (49). Another patient with emotionally unstable personality disorder treated with duloxetine, quetiapine, lamotrigine and with Dialectical Behavioral Therapy reported self-harm free and stable emotion (64). In our patient with clEDS, described above, who is suffering from general pain in the whole body, duloxetine, an antidepressant that targets pain, was dramatically effective for both pain relief and anxiety/depressiveness. This is because duloxetine has an analgesic effect, such as in fibromyalgia and other chronic pain (67, 68). For psychiatrists, the selection of effective drugs is important, as is understanding the patient's pain.

Besides pharmaceutical therapy, psychiatrists should understand and find ways to support the holistic pain of patients with EDS. In the case introduced above, our patient with cEDS had complex psychiatric symptoms, including dysthymia, dissociative identity disorder, gender dysphoria, and anorexia nervosa (12). It took years for her condition to be diagnosed by a psychiatrist, and the patient was enduring these symptoms with insufficient support from a general clinical geneticist. During those years, multiple personalities have deteriorated due to self-care for undernutrition risk brought about by anorexia nervosa and social discomfort due to gender dysphoria. We believe that the cooperation of clinical geneticists and psychiatrists can provide sufficient support to such patients.

## Conclusion

Since the lifespan of patients with EDS is not affected, psychiatrists must recognize the need for medical assistance in the course of psychological problems and mood disorders. Their pain and early disability could be the modulators of the psychiatric manifestations. To date, there is no evidence that the causative gene of EDS is expressed in the brain and that it is involved in changes of the central nervous system function. Moreover, it is difficult to assume that the symptoms of EDS are psychiatrically detailed and accurately categorized.

Patients with EDS are often disappointed and dissatisfied with not knowing the causative gene of the disease. All the causes of EDS are genetics based; although, various genes are involved in different types of EDS, which needs to be considered, given that they may have varying implications for the patient and their families. Therefore, genetic counseling and psychological support are also important (69, 70).

In addition, psychiatric care can contribute to the genetic counseling of patients with EDS and the medical management of EDS psychiatric symptoms, as well as in the development of clinical research. Both psychiatrists and clinical psychologists should be aware that these patients have a variety of physical, social, and psychological distresses associated with EDS, resulting in secondary psychiatric symptoms and in social hypoactivity.

## Author Contributions

HI and HY contributed to make diagnosis for EDS. HI and YH contributed to the writing of the manuscript. HI treats the patients' psychiatric symptoms. All authors read and approved the final manuscript.

## Conflict of Interest

The authors declare that the research was conducted in the absence of any commercial or financial relationships that could be construed as a potential conflict of interest.

## Publisher's Note

All claims expressed in this article are solely those of the authors and do not necessarily represent those of their affiliated organizations, or those of the publisher, the editors and the reviewers. Any product that may be evaluated in this article, or claim that may be made by its manufacturer, is not guaranteed or endorsed by the publisher.
